# Interconnected metabolic and reproductive signaling axis in type 2 diabetes mellitus and polyendocrine metabolic ovarian syndrome

**DOI:** 10.3389/fendo.2026.1996827

**Published:** 2026-10-20

**Authors:** Khurshid Ahmad Padder, Mohd Ashraf Ganie, Nusrat Jahan, Mohmad Aadil Yousuf, Tajali Sehar, Mudasir Ahmad Bhat, Showkat Hussain, Saba Muzaffar, Sadiq Majeed, Refaz Ahmad Dar, Touseef Sheikh

**Affiliations:** 1 Department of Endocrinology, Sher-i- Kashmir Institute of Medical Sciences, Srinagar, India; 2 Department of Biotechnology, Government Degree College (GDC) (Women), Anantnag, India; 3 Department of Life Sciences, Jaipur National University, Jaipur, Rajasthan, India; 4 Department of Allied and HealthCare, Guru Kashi University, Talwandi Sabo, Punjab, India; 5 Department of Clinical Biochemistry, Government Degree College (GDC) (Women), Anantnag, India

**Keywords:** inflammation, insulin resistance, oxidative stress, PCOS, T2DM

## Abstract

Polyendocrine metabolic ovarian syndrome (PMOS) and type 2 diabetes milletus (T2DM) are interlinked endocrine and metabolic disorders characterized by insulin resistance (IR), hyperinsulinemia and chronic low-grade inflammation. Both these abnormal physiological and metabolic conditions share overlapping cellular and molecular pathways involving insulin signaling, PI3K/Akt dysregulation, oxidative stress (OS) and inflammatory cascades. In PMOS, these disturbances further intersect with reproductive axis dysfunction including hyperandrogenism and follicular arrest. This manuscript reviews the shared metabolic and reproductive signaling pathways in PMOS and T2DM, highlighting insulin signaling defects, AMPK–mTOR imbalance, WNT/β-catenin dysregulation and inflammatory mediators. Understanding these common pathways may facilitate development of unified and more specific therapeutic strategies targeting both metabolic and reproductive dysfunction among these disorders.

## Graphical Abstract

**Figure:**
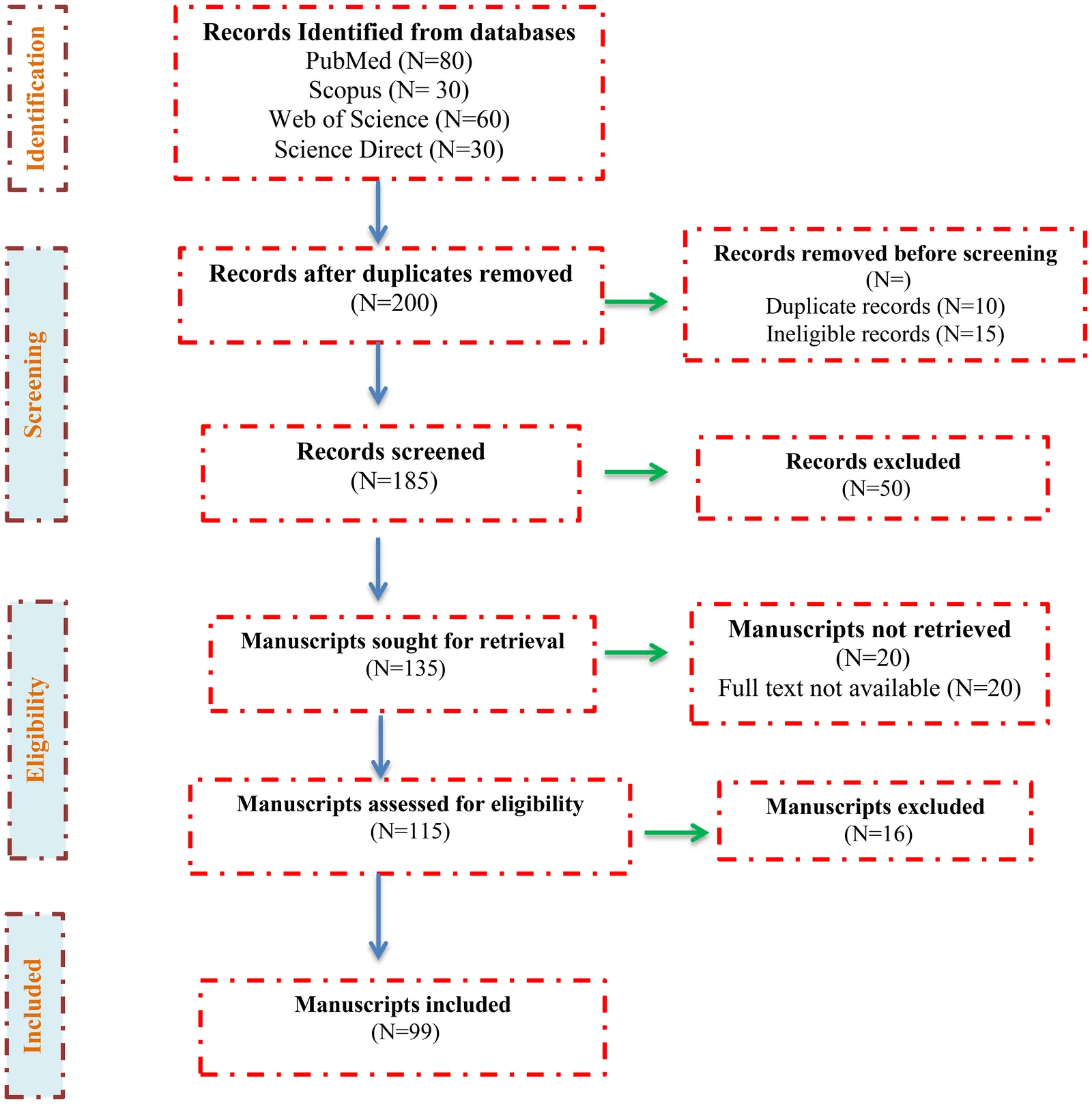
Pert diagram depicting the methodology used for this study.

## 1 Introduction

Type 2 diabetes Mellitus (T2DM) and Polyendocrine metabolic ovarian syndrome (PMOS) are among the most prevalent metabolic disorders worldwide and represent major public health concerns because of their long-term endocrine, cardiovascular and reproductive complications. T2DM is characterized by chronic hyperglycemia resulting from insulin resistance (IR) and progressive pancreatic β-cell dysfunction, whereas PMOS is a multifactorial endocrine disorder affecting women of reproductive age and is characterized by hyperandrogenism, ovulatory dysfunction and polycystic ovarian morphology (1). Increasing evidence suggests that these two disorders share several common pathogenic pathways, including insulin resistance, chronic low-grade inflammation, oxidative stress (OS), adipose tissue dysfunction and altered intracellular signaling cascades (2). Women with PMOS are at a significantly increased risk of impaired glucose tolerance and T2DM, highlighting the close metabolic association between these conditions (3, 4).

Insulin resistance is considered the central molecular defect linking PMOS and T2DM. In both disorders, impaired insulin signaling involving insulin receptor substrate-1 (IRS-1), phosphatidylinositol 3-kinase (PI3K) and protein kinase B (Akt) results in defective glucose transport and altered glycogen synthesis. Dysfunction of the PI3K/Akt pathway contributes to reduced glucose uptake in skeletal muscle and adipose tissue, ultimately leading to hyperinsulinemia and metabolic imbalance (5). In PMOS, compensatory hyperinsulinemia additionally stimulates ovarian theca cells, enhancing androgen production and worsening reproductive dysfunction (6). Simultaneously, mitogen-activated protein kinase (MAPK) signaling remains relatively preserved, promoting ovarian steroidogenesis and abnormal follicular development (7). These findings support the concept that selective IR contributes to both metabolic abnormalities and hyperandrogenism in PMOS.

Recent studies have further highlighted the importance of AMP-activated protein kinase (AMPK), mammalian target of rapamycin (mTOR), nuclear factor-kappa B (NF-κB) and WNT/β-catenin signaling pathways in the interconnected metabolic and reproductive dysfunction observed in T2DM and PMOS (7). Reduced AMPK activity impairs cellular energy sensing and favors activation of mTOR signaling, thereby contributing to insulin resistance, follicular arrest and ovarian dysfunction. Chronic activation of inflammatory mediators such as NF-κB, tumor necrosis factor-alpha (TNF-α) and interleukin-6 (IL-6) promotes persistent low-grade inflammation and OS, which exacerbate both metabolic and reproductive abnormalities (8). Dysregulation of the WNT/β-catenin pathway has also emerged as a critical contributor to impaired folliculogenesis, ovarian dysfunction and metabolic derangements (9). Adipose tissue dysfunction, mitochondrial abnormalities and oxidative stress further amplify these pathological mechanisms, creating a vicious cycle linking endocrine and metabolic disturbances (10).

Understanding the interconnected signaling axis between T2DM and PMOS has important clinical implications for early diagnosis, prevention and therapeutic intervention (3). Current therapeutic strategies primarily focus on improving insulin sensitivity and restoring metabolic balance through lifestyle modification, insulin-sensitizing agents such as metformin and hormonal therapies (11). However, emerging evidence suggests that targeting inflammatory pathways, OS and WNT signaling may provide novel therapeutic opportunities for managing both disorders simultaneously. Therefore, elucidating the shared molecular pathways underlying metabolic and reproductive dysfunction may facilitate the development of treatment strategies aimed at reducing the long term cardiometabolic and reproductive burden associated with PMOS and T2DM (11).

## 2 Methodology

A structured literature review was conducted to synthesize current evidence regarding the molecular, metabolic, inflammatory, endocrine and reproductive mechanisms linking polycystic ovary syndrome (PCOS) and type 2 diabetes mellitus (T2DM). Particular emphasis was placed on shared defects in insulin signaling, glucose and lipid metabolism, oxidative stress, chronic low-grade inflammation, adipose-tissue dysfunction and intracellular signaling pathways including PI3K/Akt, MAPK, AMPK–mTOR, NF-κB and WNT/β-catenin signaling. The review also examined the potential therapeutic relevance of these interconnected pathways. A comprehensive literature search was performed using major biomedical and multidisciplinary electronic databases, including PubMed/MEDLINE, Scopus, Web of Science, Science Direct and Google Scholar. Additional relevant publications were identified by screening the reference lists of retrieved articles and previously published literature.

The search strategy combined controlled vocabulary and free-text keywords related to PCOS, T2DM, insulin resistance and molecular signaling pathways. Representative search terms included: **“**Polycystic ovary syndrome” or “PCOS” and “Type 2 Diabetes Mellitus” or “T2DM” and “insulin resistance” or IR or “hyperinsulinemia” or “insulin signaling” or “PI3K/Akt” or “MAPK” or “AMPK” or “mTOR” or “NF-κB” or “WNT/β-catenin” or “adipokines” or “glucose metabolism”. Search terms were modified according to the indexing system and syntax of each database. Relevant literature published from database inception to August 2026 was considered.

Studies were considered eligible when they provided information relevant to the metabolic, endocrine, inflammatory, oxidative or molecular relationship between PCOS and T2DM.

### 2.1 Inclusion criteria

Inclusion criteria were:

1. Original research studies, systematic reviews, meta-analyses and relevant narrative reviews.
2. Studies investigating PCOS, T2DM or their metabolic association.
3. Studies addressing insulin resistance, hyperinsulinemia, glucose or lipid metabolism.
4. Studies examining molecular pathways relevant to PCOS and/or T2DM, including PI3K/Akt, MAPK, AMPK–mTOR, NF-κB and WNT/β-catenin signaling.
5. Human, animal and relevant *in-vitro* experimental studies providing mechanistic evidence.
6. Articles published in peer-reviewed journals.

### 2.2 Exclusion criteria

Exclusion criteria were:

1. Duplicate publications.
2. Conference abstracts without sufficient supporting data.
3. Articles unrelated to the metabolic or molecular relationship between PCOS and T2DM.
4. Non-peer-reviewed material, opinion pieces and non-scientific reports.
5. Studies for which the available information was insufficient to establish relevance to the objectives of the review.

Retrieved records were initially screened on the basis of their titles and abstracts. Articles considered potentially relevant were subsequently evaluated through full-text assessment. Publications fulfilling the predefined eligibility criteria were included in the final qualitative synthesis. Reference-list screening of key articles was additionally performed to identify relevant studies that might not have been retrieved through the primary database search.

The literature identification and selection process was documented to enhance transparency and reproducibility. The overall workflow comprised database searching, removal of duplicate records, title/abstract screening, full-text assessment, eligibility determination and final qualitative synthesis. Where appropriate, the reporting structure was informed by the PRISMA 2020 framework.

## 3 Review of literature

### 3.1 Insulin signaling pathway dysfunction

Insulin signaling is a highly coordinated molecular cascade essential for maintaining glucose homeostasis, cellular growth and metabolic regulation. The process is initiated when insulin binds to its receptor, a transmembrane tyrosine kinase, leading to receptor autophosphorylation and activation of downstream signaling pathways (12). In metabolic disorders such as T2DM and PMOS, defects in this pathway result in IR, a hallmark feature linking both conditions.

One of the earliest abnormalities in IR is impaired insulin receptor signaling. Under normal conditions, insulin binding triggers tyrosine phosphorylation of the receptor and recruitment of intracellular adaptor proteins such as insulin receptor substrates (13, 14). However, in IR states, reduced receptor kinase activity or increased inhibitory signaling diminishes receptor autophosphorylation, thereby weakening downstream signal transduction. Genetic mutations or post-receptor defects further exacerbate this impairment, contributing to decreased insulin sensitivity.

A critical step in insulin signaling involves phosphorylation of IRS-1 and IRS-2, which act as docking proteins for downstream effectors. Tyrosine phosphorylation of IRS proteins promotes signal propagation, whereas serine phosphorylation often induced by inflammatory cytokines, free fatty acids, or OS impairs their function (7). This shift from activating to inhibitory phosphorylation is a central driver underlying IR in both T2DM and PMOS (15). Reduced IRS-1/IRS-2 activity leads to diminished recruitment of phosphatidylinositol 3-kinase (PI3K), thereby disrupting metabolic signaling.

The PI3K/Akt pathway is the principal mediator of insulin’s metabolic effects, including glucose uptake, glycogen synthesis and inhibition of gluconeogenesis. Upon activation, PI3K generates phosphatidylinositol-3,4,5-trisphosphate (PIP3), which recruits and activates Akt, a key serine/threonine kinase (16). Akt then facilitates translocation of GLUT4 to the plasma membrane, enabling glucose uptake in insulin-sensitive tissues. In IR, defective PI3K/Akt activation results in reduced glucose transport and metabolic dysregulation (17). Experimental inhibition of PI3K has been shown to abolish insulin stimulated glucose uptake, highlighting its essential role in metabolic control.

Interestingly, insulin signaling bifurcates into two major branches: the metabolic PI3K/Akt pathway and the MAPK pathway. While the PI3K/Akt arm is selectively impaired in IR, the MAPK pathway responsible for cell growth and proliferation remains relatively preserved (14). This phenomenon, termed selective IR, explains why metabolic actions of insulin (e.g., glucose uptake) are compromised, whereas mitogenic effects (e.g., cell proliferation and androgen synthesis in ovarian theca cells) are maintained or even enhanced. In PMOS, this selective signaling contributes to hyperandrogenism, while in T2DM, it promotes vascular complications and abnormal cell growth.

Collectively, insulin signaling dysfunction involves a combination of receptor level defects, impaired IRS phosphorylation, reduced PI3K/Akt activation and preservation of MAPK signaling (18). These alterations disrupt metabolic homeostasis while maintaining or exaggerating growth related pathways, thereby linking metabolic and reproductive abnormalities observed in PMOS and T2DM. Understanding these molecular defects provides a basis for targeted therapeutic interventions, such as insulin sensitizers that restore PI3K/Akt signaling without overstimulating mitogenic pathways (**Figures 1A–C**).

**Figure 1:**
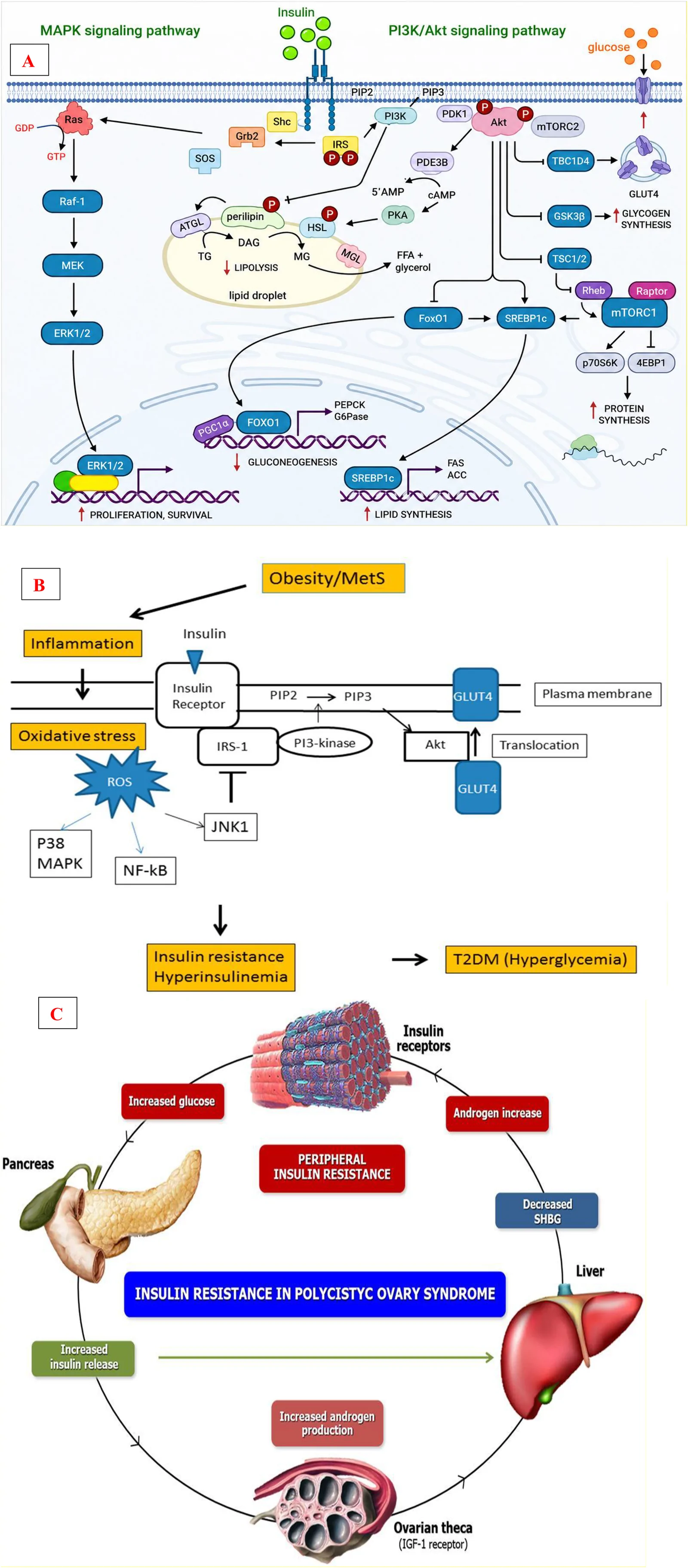
**(A–C)** insulin signaling defects in PMOS and T2DM.

### 3.2 Hyperinsulinemia and hyperandrogenism axis

Hyperinsulinemia represents a central pathophysiological driver linking metabolic dysfunction to reproductive abnormalities in PMOS. Elevated circulating insulin levels, secondary to IR exert direct and indirect effects on ovarian steroidogenesis. At the ovarian level, insulin enhances androgen biosynthesis by stimulating theca cell activity, primarily through amplification of steroidogenic enzyme expression such as CYP17A1 and by potentiating insulin-like growth factor-1 (IGF-1) signaling pathways. This results in increased production of androgens including testosterone and androstenedione (4, 19). Concurrently, insulin suppresses hepatic synthesis of sex hormone-binding globulin (SHBG), thereby increasing the bioavailability of free testosterone and exacerbating hyperandrogenemia (20). Importantly, insulin acts synergistically with LH to further augment androgen production in ovarian theca cells, amplifying gonadotropin driven steroidogenesis and disrupting normal follicular maturation. This cooperative interaction contributes to follicular arrest, anovulation and the characteristic ovarian dysfunction observed in PMOS (21, 22). Collectively, the hyperinsulinemia–hyperandrogenism axis constitutes a critical mechanistic link between metabolic and reproductive disturbances, reinforcing the bidirectional relationship between IR and ovarian dysfunction.

### 3.3 AMPK–mTOR signaling axis

The AMP-activated protein kinase (AMPK) mechanistic target of rapamycin (mTOR) signaling axis plays a pivotal role in integrating cellular energy status with metabolic and reproductive function and its dysregulation is increasingly recognized in both T2DM and PMOS. AMPK functions as a cellular energy sensor that is activated under conditions of increased AMP/ATP ratio, promoting catabolic pathways and inhibiting anabolic processes to restore energy balance. In IR states, reduced AMPK activity impairs glucose uptake, fatty acid oxidation and mitochondrial function, thereby exacerbating metabolic dysfunction (23, 24). Conversely, mTOR, a central regulator of cell growth and protein synthesis, is often hyperactivated in these conditions due to nutrient excess and chronic hyperinsulinemia. Enhanced mTOR signaling contributes to IR through negative feedback inhibition of insulin receptor substrate (13) proteins and disrupts normal ovarian physiology by promoting aberrant follicular growth and steroidogenesis (25, 26). In the ovary, excessive mTOR activity has been linked to follicular arrest and hyperandrogenism, key features of PMOS pathogenesis. Importantly, the pharmacological agent Metformin exerts its therapeutic effects largely through activation of AMPK, leading to suppression of mTOR signaling, improvement in insulin sensitivity and restoration of ovulatory function (27, 28). Thus, the imbalance between diminished AMPK activity and enhanced mTOR signaling represents a critical mechanistic nexus linking metabolic derangements with reproductive dysfunction in both T2DM and PMOS (**Figures 2A–C**).

**Figure 2:**
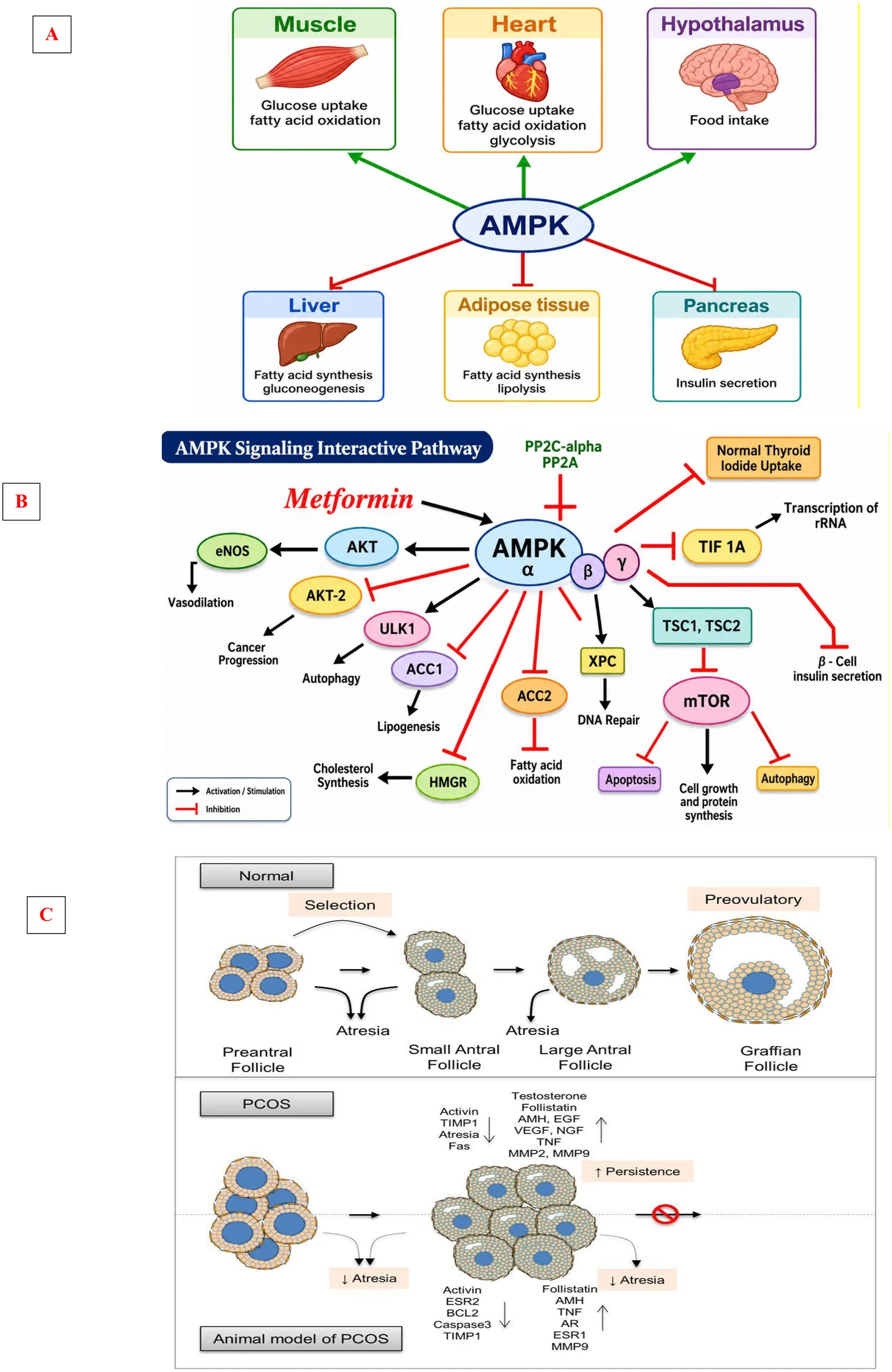
**(A– C)** AMPK–mTOR pathway in metabolic and reproductive dysfunction.

### 3.4 Inflammation and OS pathways

Chronic low-grade inflammation and OS are fundamental mechanisms linking obesity, IR and metabolic disorders such as T2DM, PMOS, CVD and metabolic syndrome. Persistent metabolic stress caused by excess nutrients, hyperglycemia and dyslipidemia activates several intracellular signaling pathways that promote inflammatory cytokine production and reactive oxygen species (ROS) generation. These processes create a vicious cycle in which inflammation enhances OS, while OS further amplifies inflammatory signaling, ultimately resulting in cellular dysfunction, IR, mitochondrial damage and disease progression (29, 30).

### 3.5 Activation of NF-κB signaling

Among the molecular pathways involved in inflammation, the nuclear factor-kappa B (NF-κB) signaling pathway serves as a master regulator of inflammatory responses. Under physiological conditions, NF-κB remains inactive in the cytoplasm through its association with inhibitory proteins known as IκBs. Exposure to inflammatory stimuli such as hyperglycemia, free fatty acids, advanced glycation end-products (AGEs), oxidative stress and inflammatory cytokines activates the IκB kinase (31) complex, leading to phosphorylation and degradation of IκB proteins. This permits NF-κB to translocate into the nucleus, where it promotes transcription of numerous pro-inflammatory genes including tumor necrosis factor-alpha (TNF-α), interleukin-6 (IL-6), interleukin-1β (IL-1β), cyclooxygenase-2 (COX-2), inducible nitric oxide synthase (32) and various chemokines (33).

In obesity and IR states, enlarged adipocytes release excessive free fatty acids that activate Toll-like receptor-4 (TLR4), initiating NF-κB signaling through the MyD88-dependent pathway. Simultaneously, infiltrating macrophages within adipose tissue contribute additional inflammatory mediators, further sustaining NF-κB activation. Persistent activation of this pathway interferes with insulin signaling by promoting serine phosphorylation of insulin receptor substrate-1 (IRS-1), thereby impairing glucose uptake and insulin sensitivity (29, 34).

NF-κB activation also contributes significantly to endothelial dysfunction and vascular inflammation. Increased expression of adhesion molecules such as vascular cell adhesion molecule-1 (VCAM-1) and intracellular adhesion molecule-1 (ICAM-1) facilitates leukocyte recruitment into vascular tissues, promoting atherosclerosis and cardiovascular complications frequently observed in metabolic diseases. Furthermore, NF-κB stimulates transcription of several genes involved in OS, creating a positive feedback loop between inflammation and ROS production (33).

### 3.6 Increased production of pro-inflammatory cytokines

Activation of NF-κB results in increased production of numerous inflammatory cytokines, among which TNF-α and IL-6 play central pathogenic roles. TNF-α, primarily secreted by adipose tissue macrophages and dysfunctional adipocytes, is one of the earliest cytokines implicated in obesity-induced IR. It inhibits insulin receptor signaling by inducing serine phosphorylation of IRS proteins and suppressing glucose transporter-4 (GLUT4) expression, thereby reducing glucose uptake in skeletal muscle and adipose tissue (35).

TNF-α additionally promotes lipolysis, increasing circulating free fatty acid concentrations that further activate inflammatory pathways and worsen IR. Elevated TNF-α levels have consistently been reported in patients with obesity, T2DM, metabolic syndrome and PMOS, indicating its important contribution to chronic metabolic inflammation (29).

IL-6 is another multifunctional cytokine produced by adipocytes, macrophages, endothelial cells and skeletal muscle. Although transient increases in IL-6 during exercise exert anti-inflammatory effects, chronically elevated IL-6 contributes to metabolic dysfunction by stimulating hepatic glucose production, suppressing insulin receptor signaling and promoting hepatic synthesis of acute-phase proteins, particularly CRP. Elevated serum IL-6 concentrations correlate strongly with obesity, IR and increased cardiovascular risk (36).

CRP, synthesized primarily by hepatocytes under IL-6 stimulation, serves as an important biomarker of systemic inflammation. High-sensitivity CRP (hs-CRP) has emerged as an independent predictor of CVD, T2DM and metabolic syndrome. Elevated CRP levels reflect persistent activation of innate immunity and endothelial dysfunction. Beyond being a marker, CRP may directly contribute to vascular inflammation by enhancing monocyte recruitment, complement activation and endothelial injury (37). Collectively, elevated TNF-α, IL-6 and CRP establish a chronic inflammatory state that perpetuates IR, β-cell dysfunction, endothelial damage and accelerated progression of metabolic disorders. Numerous clinical studies have demonstrated positive correlations between these inflammatory biomarkers and disease severity, emphasizing their utility as both prognostic indicators and potential therapeutic targets.

### 3.7 Reactive oxygen species accumulation and mitochondrial dysfunction

OS occurs when intracellular ROS production exceeds the antioxidant defense capacity of the cell. Major ROS include superoxide anion (O_2_•−), hydrogen peroxide (H_2_O_2_), hydroxyl radicals (•OH) and peroxynitrite (ONOO−). Physiologically, ROS participate in cellular signaling, immune defense and regulation of gene expression. However, excessive ROS production causes oxidative damage to proteins, lipids, DNA and mitochondria (31).

Hyperglycemia, elevated free fatty acids, chronic inflammation and mitochondrial electron transport chain dysfunction represent the principal sources of ROS generation in metabolic diseases. Increased glucose oxidation overwhelms mitochondrial oxidative phosphorylation, resulting in electron leakage from complexes I and III of the electron transport chain and excessive superoxide production. Simultaneously, activation of NADPH oxidase enzymes and xanthine oxidase further amplifies intracellular ROS generation (38).

Mitochondria are both major producers and primary targets of ROS. Excessive ROS damages mitochondrial DNA (mtDNA), oxidizes membrane phospholipids, disrupts respiratory chain complexes and reduces ATP production. Loss of mitochondrial membrane potential facilitates opening of the mitochondrial permeability transition pore, resulting in cytochrome c release and activation of apoptotic pathways. Progressive mitochondrial dysfunction further increases ROS generation, establishing a self-perpetuating cycle of oxidative damage (39).

ROS accumulation also activates stress-sensitive signaling pathways including NF-κB, c-Jun N-terminal kinase (JNK) and p38 mitogen-activated protein kinase (MAPK), thereby enhancing inflammatory cytokine production and IR. Activation of JNK promotes inhibitory serine phosphorylation of IRS-1, disrupting insulin signaling and glucose homeostasis. Consequently, oxidative stress and inflammation synergistically impair metabolic regulation and contribute to disease progression (40).

To counteract OS, cells possess endogenous antioxidant systems including superoxide dismutase (41), catalase (CAT), glutathione peroxidase (GPx), glutathione (42) and transcription factor nuclear factor erythroid 2-related factor 2 (Nrf2). Under chronic metabolic stress, however, these antioxidant defenses become overwhelmed or depleted, permitting persistent oxidative damage and mitochondrial dysfunction. Reduced antioxidant enzyme activity has been consistently reported in patients with obesity, T2DM, PMOS and CVD, supporting the central role of oxidative stress in metabolic pathophysiology (43).

Overall, activation of NF-κB signaling, increased production of inflammatory cytokines including TNF-α, IL-6 and CRP, together with ROS accumulation and mitochondrial dysfunction, constitute interconnected molecular pathways that drive chronic inflammation and metabolic dysfunction. These mechanisms reinforce one another through multiple positive feedback loops, promoting IR, endothelial dysfunction, β-cell failure and tissue injury. Consequently, therapeutic strategies targeting inflammation, OS and mitochondrial health have emerged as promising approaches for preventing and managing metabolic diseases.

### 3.8 WNT/β-catenin signaling dysregulation

The WNT/β-catenin signaling pathway is an evolutionarily conserved signaling cascade that regulates embryonic development, cell proliferation, differentiation, apoptosis and tissue homeostasis. In the ovary, canonical WNT signaling plays a crucial role in follicular development, granulosa cell proliferation, oocyte maturation and steroidogenesis. Growing evidence indicates that dysregulation of this pathway contributes significantly to the reproductive and metabolic abnormalities observed in PMOS. Aberrant WNT/β-catenin signaling has been implicated in impaired folliculogenesis, granulosa cell apoptosis, abnormal adipocyte differentiation, IR and chronic inflammation, thereby linking ovarian dysfunction with systemic metabolic disturbances. Recent molecular studies further suggest that WNT signaling interacts with several metabolic pathways, including PI3K/Akt, AMPK, mTOR, TGF-β/Smad and Hippo signaling, highlighting its central role in the pathogenesis of PMOS.

### 3.9 Altered folliculogenesis in PMOS

Normal ovarian folliculogenesis requires tightly regulated communication between oocytes, granulosa cells and theca cells. Canonical WNT signaling is initiated when WNT ligands bind to Frizzled (FZD) receptors and LRP5/6 co-receptors, leading to stabilization and nuclear translocation of β-catenin, which subsequently activates transcription of genes involved in cell proliferation and differentiation. In healthy ovaries, this pathway promotes granulosa cell survival, follicular growth, cumulus expansion and ovulation. Conversely, in PMOS, abnormal activation or suppression of WNT signaling disrupts these physiological processes, resulting in arrested follicular development and accumulation of immature antral follicles, one of the defining morphological features of the syndrome (44).

Experimental studies have demonstrated altered expression of WNT ligands, β-catenin, glycogen synthase kinase-3β (GSK-3β) and downstream transcription factors in granulosa cells isolated from women with PMOS. Increased GSK-3β activity enhances β-catenin degradation, thereby suppressing transcription of genes essential for follicular maturation. Furthermore, dysregulated β-catenin signaling promotes granulosa cell apoptosis and impairs responsiveness to follicle-stimulating hormone (FSH), ultimately leading to follicular arrest and chronic anovulation. Hyperandrogenism, a hallmark of PMOS, further disturbs WNT signaling by altering granulosa-theca cell communication and enhancing oxidative stress within the ovarian microenvironment. These abnormalities collectively impair oocyte competence and reduce fertility potential (44, 45). In addition to follicular maturation, WNT signaling also regulates ovarian steroidogenesis through interactions with luteinizing hormone (LH)-mediated pathways. Aberrant β-catenin activation influences the expression of steroidogenic enzymes such as CYP17A1 and StAR, promoting excessive androgen production by theca cells. Elevated androgen levels subsequently inhibit normal follicular selection, creating a vicious cycle that perpetuates ovarian dysfunction in PMOS (46).

### 3.10 Links to adipogenesis and insulin sensitivity

Beyond ovarian physiology, WNT/β-catenin signaling is a major regulator of adipose tissue development and systemic glucose metabolism. Canonical WNT signaling inhibits adipogenesis by suppressing the expression of key adipogenic transcription factors including peroxisome proliferator-activated receptor-γ (PPARγ) and CCAAT/enhancer-binding protein-α (C/EBPα). During normal adipocyte differentiation, reduced WNT activity permits precursor cells to mature into insulin-sensitive adipocytes capable of efficient lipid storage and endocrine regulation. In PMOS, disruption of this balance contributes to adipose tissue dysfunction characterized by adipocyte hypertrophy, ectopic lipid accumulation, chronic inflammation and altered adipokine secretion (10).

Abnormal adipogenesis contributes directly to IR, one of the major metabolic abnormalities affecting approximately 50–80% of women with PMOS. Impaired WNT signaling reduces insulin-stimulated glucose uptake by altering expression of glucose transporter-4 (GLUT4) and disrupting downstream insulin signaling pathways. Dysfunctional adipose tissue also secretes increased amounts of pro-inflammatory cytokines such as TNF-α, IL-6 and MCP-1, together with reduced adiponectin production, thereby aggravating IR and systemic inflammation. These metabolic disturbances further enhance ovarian androgen synthesis through compensatory hyperinsulinemia, creating a reciprocal relationship between reproductive and metabolic dysfunction (47).

Accumulating evidence indicates that WNT inhibitors such as Dickkopf-1 (DKK1) and secreted frizzled-related proteins (SFRPs) are dysregulated in obesity and IR states. Elevated circulating concentrations of these inhibitory molecules have been associated with impaired β-catenin signaling, excessive adipocyte differentiation, increased visceral adiposity and reduced insulin sensitivity. Since obesity amplifies IR and hyperandrogenism in PMOS, altered expression of WNT antagonists may represent an important mechanistic link between adipose tissue dysfunction and ovarian pathology (47) as depicted in **Figure 3**.

**Figure 3:**
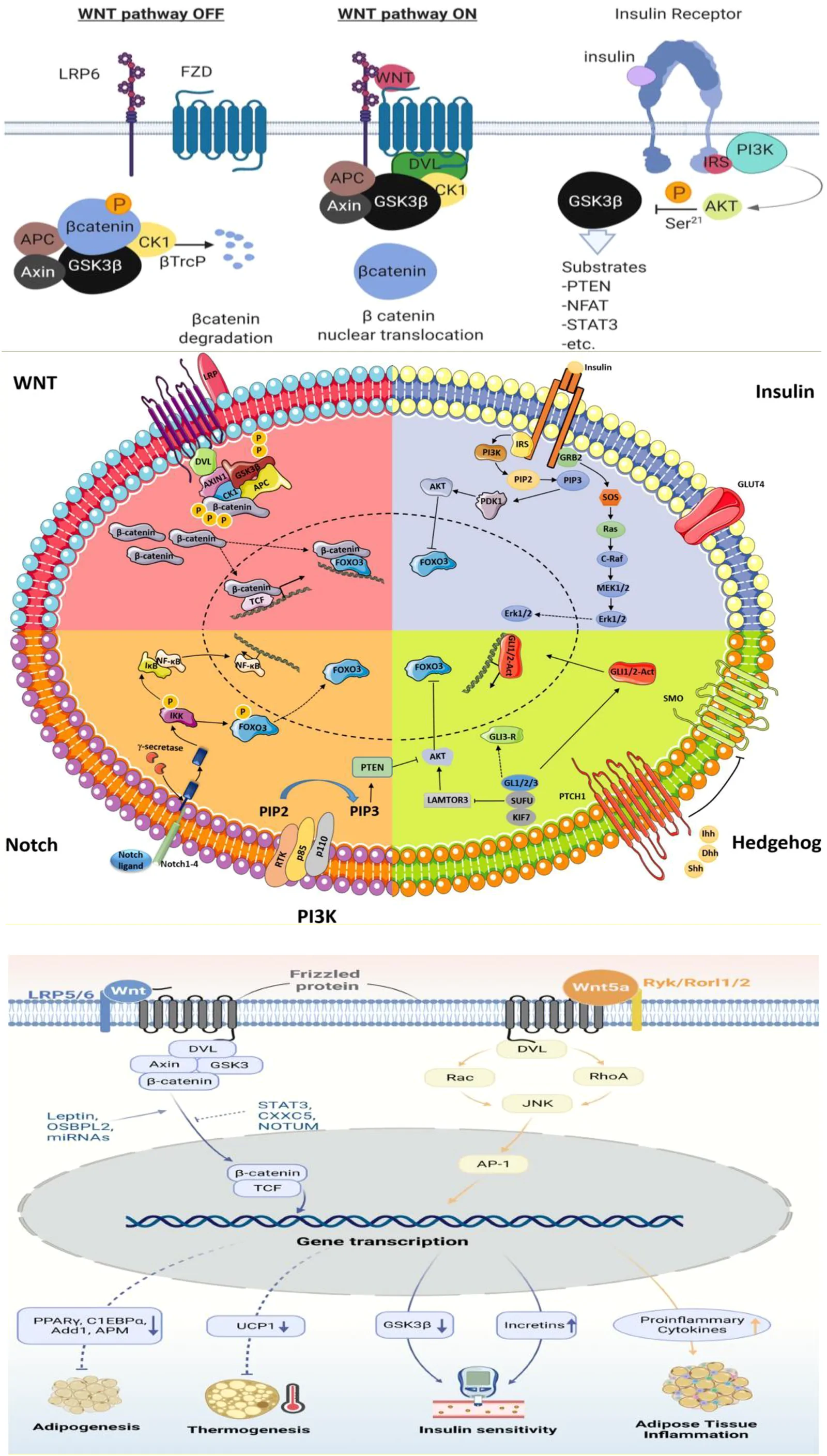
WNT/β-catenin signalling in ovarian and metabolic regulation.

### 3.11 Hypothalamic–pituitary–ovarian axis dysregulation

Dysregulation of the hypothalamic–pituitary–ovarian (HPO) axis is a central neuroendocrine abnormality in women with PMOS, characterized primarily by increased gonadotropin-releasing hormone (GnRH) pulse frequency and amplitude. Altered hypothalamic sensitivity to steroid hormone feedback, particularly reduced responsiveness to progesterone-mediated inhibition, leads to persistent rapid GnRH pulsatility. This preferentially stimulates luteinizing hormone (LH) secretion over FSH, contributing to an elevated LH/FSH ratio commonly observed in PMOS patients. Elevated LH enhances ovarian theca cell androgen production, which further perpetuates hyperandrogenism and disrupts normal ovarian cyclicity. Neuroendocrine studies have shown that impaired feedback regulation involving kisspeptin neurons, γ-aminobutyric acid (GABA) and anti-Müllerian hormone (AMH) may contribute significantly to this altered GnRH secretion pattern, suggesting a multifactorial mechanism underlying HPO axis dysfunction in PMOS (48–50).

A hallmark consequence of this neuroendocrine imbalance is the elevated LH/FSH ratio, which contributes directly to abnormal folliculogenesis and impaired ovulation. While LH promotes androgen synthesis by ovarian theca cells, relatively insufficient FSH impairs granulosa cell aromatase activity, limiting the conversion of androgens to estrogens. This hormonal imbalance disrupts dominant follicle selection and arrests follicular development at the small antral stage, resulting in the characteristic polycystic ovarian morphology. Studies have demonstrated that chronic exposure to elevated LH and androgens leads to premature granulosa cell differentiation and reduced follicular responsiveness to FSH, ultimately preventing ovulation and causing menstrual irregularities. Furthermore, elevated AMH levels produced by accumulated small follicles may further inhibit FSH sensitivity and contribute to sustained follicular arrest, creating a vicious cycle of ovarian dysfunction (51–53).

Emerging evidence suggests that HPO axis dysregulation in PMOS is closely linked with metabolic disturbances, particularly IR and hyperinsulinemia, which can amplify neuroendocrine abnormalities. Insulin may enhance pituitary LH secretion and sensitize ovarian theca cells to LH, thereby exacerbating androgen excess and further impairing follicular maturation. Additionally, chronic inflammation and altered adipokine signaling may affect hypothalamic neuronal networks regulating GnRH pulsatility. These interactions highlight the bidirectional relationship between metabolic dysfunction and reproductive endocrine abnormalities in PMOS. Therapeutic strategies targeting HPO axis restoration such as lifestyle interventions, insulin sensitizers and ovulation induction agents have shown effectiveness in improving ovulatory function, emphasizing the clinical importance of understanding HPO dysregulation in disease pathogenesis (4, 54).

### 3.12 Adipokines and metabolic crosstalk

Adipokines play a central role in mediating the metabolic and reproductive abnormalities observed in insulin-resistant states such as obesity and PMOS. Among these, adiponectin is a key insulin-sensitizing adipokine secreted predominantly by adipose tissue. Reduced circulating adiponectin levels have consistently been associated with increased IR, impaired glucose utilization and chronic low-grade inflammation. Adiponectin enhances insulin sensitivity through activation of AMP-activated protein kinase (AMPK) and promotion of fatty acid oxidation, thereby improving glucose homeostasis. Several studies have demonstrated significantly decreased adiponectin concentrations in women with PMOS, independent of obesity, suggesting its direct contribution to the pathogenesis of metabolic dysfunction in these patients. Lower adiponectin levels are also linked with hyperandrogenism and adverse cardiovascular risk profiles, highlighting its importance in the metabolic crosstalk between adipose tissue and ovarian function (55–57) as depicted in **Figure 4**.

**Figure 4:**
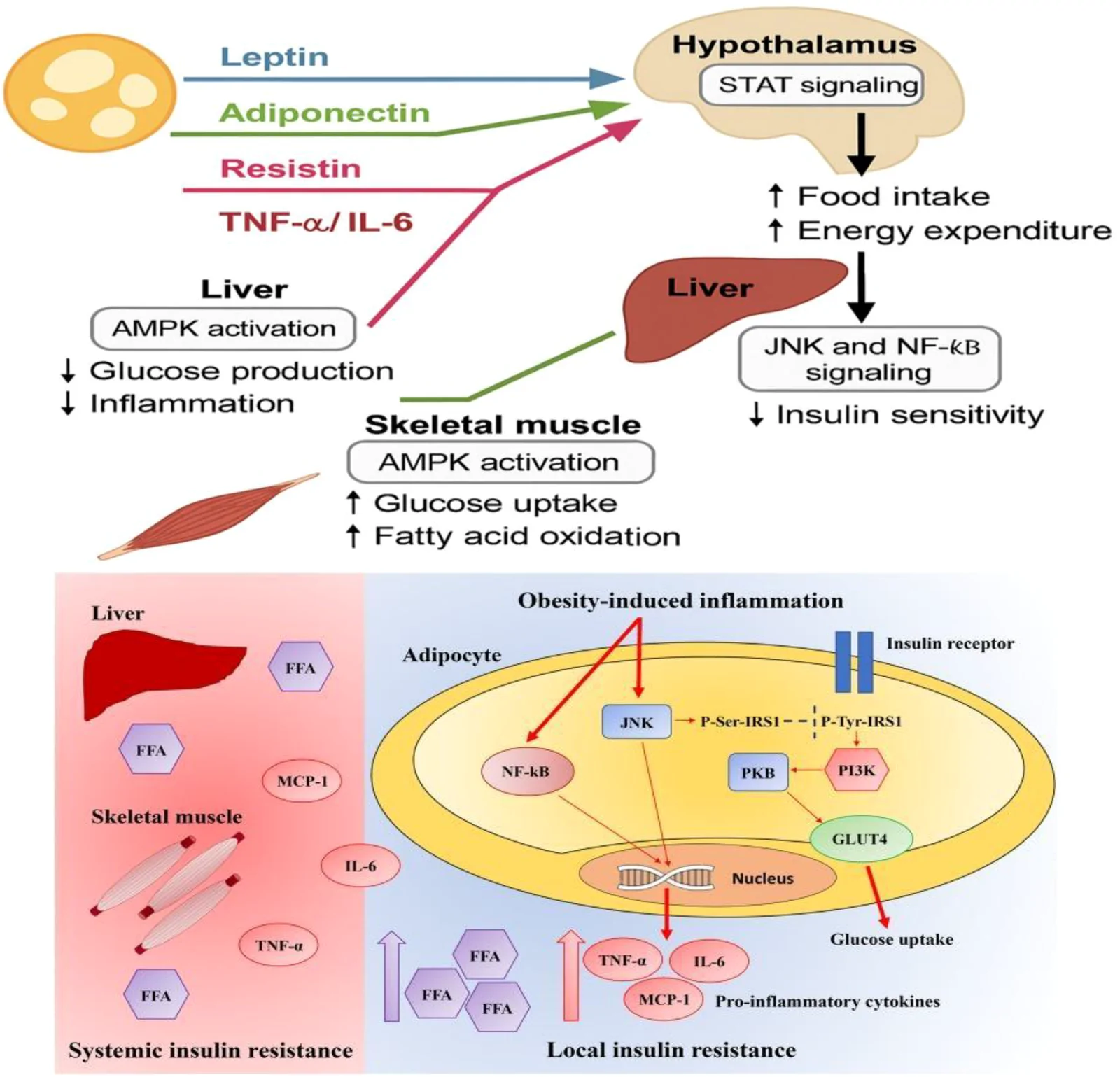
Adipokine-mediated metabolic-reproductive interaction.

Leptin, another major adipokine produced by adipocytes, is involved in the regulation of appetite, energy expenditure and reproductive endocrinology. Elevated leptin levels are commonly observed in obesity and PMOS due to leptin resistance, a condition in which target tissues fail to respond adequately to circulating leptin. Hyperleptinemia has been shown to disrupt hypothalamic–pituitary–gonadal (HPG) axis signaling, thereby impairing gonadotropin secretion and ovarian follicular development. In the ovary, leptin may directly inhibit steroidogenesis and interfere with normal ovulation, contributing to anovulation and infertility. Furthermore, leptin promotes inflammatory cytokine production and oxidative stress, thereby exacerbating IR and reproductive dysfunction. Clinical studies have reported positive correlations between serum leptin levels, body mass index (BMI), IR and androgen excess in women with PMOS, emphasizing the role of leptin in metabolic cum reproductive crosstalk (58–60).

Resistin is a pro-inflammatory adipokine implicated in the development of IR and chronic metabolic inflammation. Initially identified as a link between obesity and T2DM, resistin promotes the expression of inflammatory mediators such as TNF-α and IL-6, which impair insulin signaling pathways. Elevated resistin levels have been reported in individuals with obesity, metabolic syndrome and PMOS, where they correlate positively with markers of inflammation and IR. Resistin-mediated inflammation contributes to endothelial dysfunction, altered glucose metabolism and ovarian dysfunction, thereby reinforcing the pathogenic interaction between adipose tissue and reproductive abnormalities. Moreover, increased resistin expression in adipose tissue macrophages suggests its role in sustaining chronic low-grade inflammation characteristic of metabolic disorders. Collectively, these findings support the involvement of resistin in the inflammatory and metabolic disturbances underlying IR reproductive disorders (61–63) as described in **Figure 5**.

**Figure 5:**
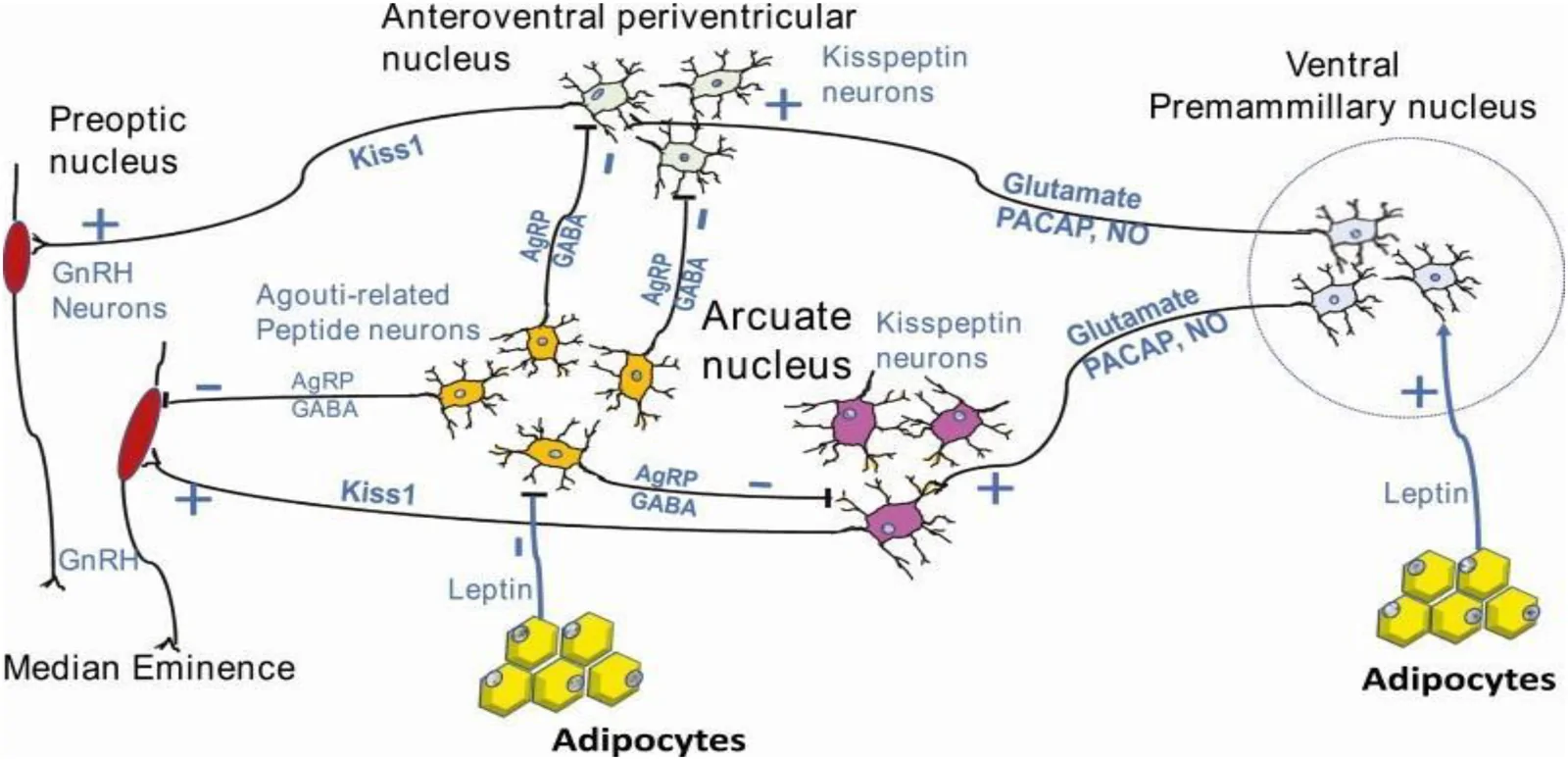
Integrated pathophysiological model.

### 3.13 Shared signaling pathways in PMOS and T2DM mellitus

PCOS and T2DM share several molecular and metabolic abnormalities that contribute to IR, chronic inflammation, endocrine dysfunction and altered cellular signaling. Increasing evidence suggests that both disorders are interconnected through common intracellular pathways including PI3K/Akt, MAPK, AMPK, mTOR, NF-κB and WNT signaling cascades. These pathways regulate glucose metabolism, ovarian steroidogenesis, follicular development, cellular proliferation and inflammatory responses. Dysregulation of these signaling networks contributes to the pathophysiology of impaired glucose uptake, IR, hyperinsulinemia, hyperandrogenism and metabolic syndrome commonly observed in women with PMOS and patients with T2DM (4, 64–66) as depicted in **Figure 6**.

**Figure 6:**
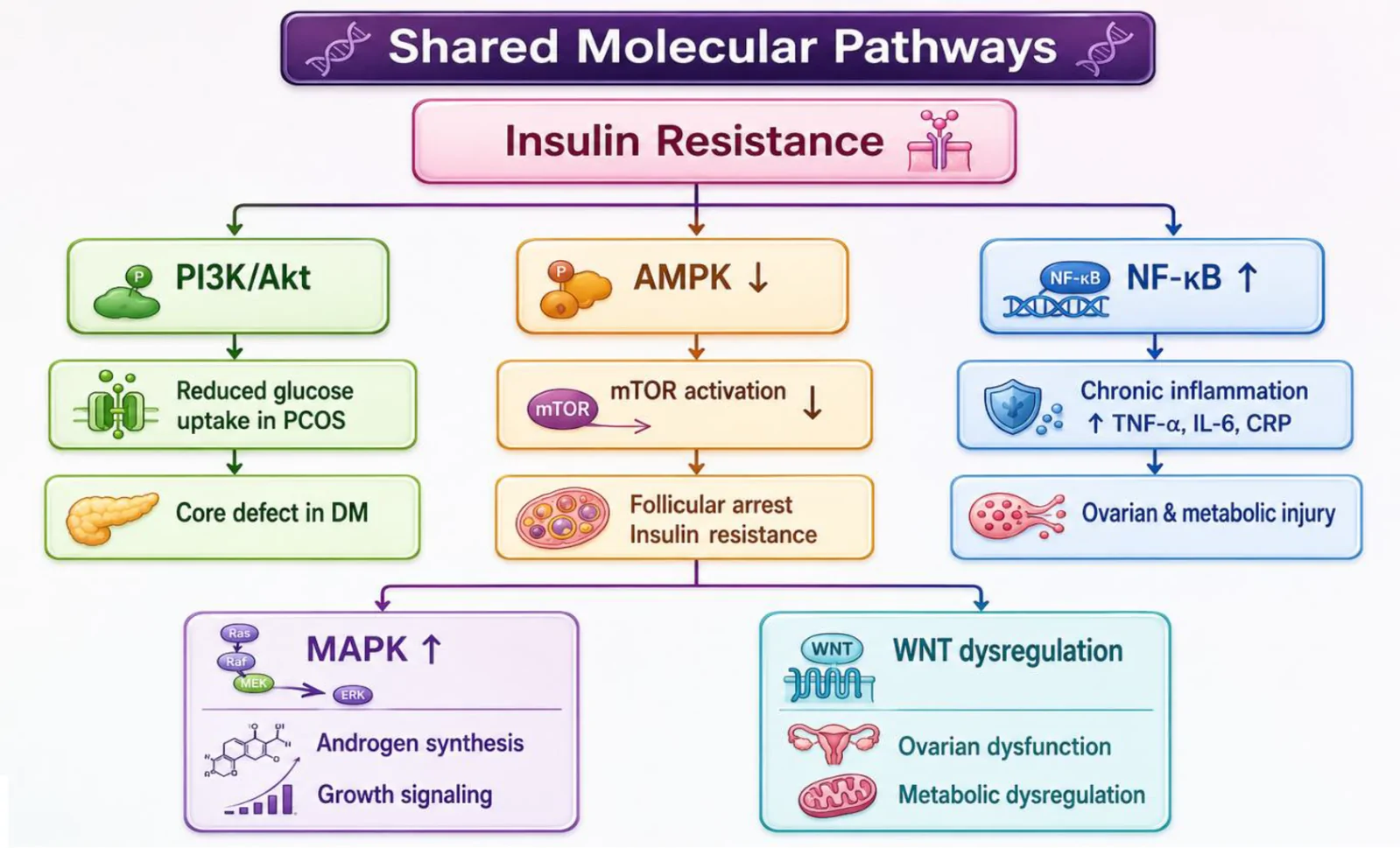
Shared signaling pathways in PMOS and T2DM.

The PI3K/Akt pathway plays a central role in insulin signaling and glucose homeostasis. In healthy individuals, insulin binding activates PI3K and downstream Akt phosphorylation, promoting GLUT4 translocation and glucose uptake. In PMOS, defects in PI3K/Akt signaling impair insulin-mediated glucose transport, thereby contributing to systemic IR and compensatory hyperinsulinemia. Similar abnormalities are considered the core molecular defect in T2DM, where impaired Akt activation leads to reduced peripheral glucose utilization (67, 68). Hyperinsulinemia further stimulates ovarian theca cells to produce excess androgens, aggravating reproductive dysfunction in PMOS (69).

The MAPK pathway is another important signaling cascade implicated in both disorders. In PMOS, MAPK signaling contributes to ovarian steroidogenesis and excessive androgen synthesis by enhancing the activity of steroidogenic enzymes in ovarian theca cells (70). Elevated insulin and luteinizing hormone levels activate MAPK signaling, thereby promoting hyperandrogenism and follicular abnormalities. In T2DM, MAPK signaling is primarily associated with cellular growth, proliferation, OS and vascular complications (65, 71). Chronic activation of this pathway contributes to endothelial dysfunction and diabetic complications, including nephropathy and CVD. Thus, MAPK signaling represents a crucial link between metabolic and reproductive dysfunctions.

AMP-activated protein kinase (AMPK) functions as a cellular energy sensor and plays an important role in regulating glucose and lipid metabolism and insulin sensitivity. Reduced or dysregulated AMPK activity has been implicated in PMOS, where AMPK suppression may contribute to altered steroidogenesis, impaired ovulatory function and metabolic dysfunction (10). In metabolic disease, AMPK activation promotes glucose uptake and fatty-acid oxidation and counteracts excessive anabolic signaling through mTORC1 (72). Metformin, widely used in T2DM and also used in selected women with PMOS, influences AMPK-dependent and AMPK-independent mechanisms and can improve insulin sensitivity and several metabolic and reproductive features of PMOS (73, 74). The mTOR pathway is closely interconnected with AMPK and PI3K/Akt signaling and participates in nutrient sensing, cellular growth, protein synthesis and reproductive physiology. Dysregulation of the AMPK–mTOR axis has therefore been proposed as a contributor to metabolic and reproductive abnormalities in PMOS and T2DM (72, 75).

Chronic low-grade inflammation is another important feature shared by PMOS and T2DM and is closely linked to NF-κB signaling. NF-κB can be activated by oxidative stress, inflammatory cytokines, obesity-associated metabolic stress and other stimuli, resulting in increased expression of inflammatory mediators such as TNF-α and IL-6 (76, 77). In PMOS, NF-κB signaling has been implicated in IR, hyperandrogenism, cardiovascular risk and reproductive/endometrial dysfunction (77). In T2DM, inflammatory signaling involving IKK/NF-κB contributes to impaired insulin action and metabolic dysfunction (7, 76). Thus, persistent NF-κB activation provides an important molecular link between inflammation and IR in both disorders.

WNT signaling in polycystic ovary syndrome (PCOS) comprises both canonical β-catenin-dependent and non-canonical β-catenin-independent pathways. The canonical pathway is initiated by binding of WNT ligands to Frizzled (FZD) receptors and LRP5/6 co-receptors, resulting in inhibition of the β-catenin destruction complex and stabilization and nuclear accumulation of β-catenin. Nuclear β-catenin interacts with T-cell factor/lymphoid enhancer factor (TCF/LEF) transcription factors to regulate genes involved in cell proliferation, differentiation, follicular development and steroidogenic processes (78, 79). Dysregulation of this pathway has therefore been implicated in altered folliculogenesis and ovarian dysfunction in PCOS (80, 81). In contrast, non-canonical WNT signaling is largely β-catenin-independent and includes the planar cell polarity and WNT/Ca²^+^ pathways, which regulate cytoskeletal organization, cellular polarity, intracellular calcium signaling and transcriptional responses (82, 83). Although these pathways may contribute to ovarian cellular function, their specific contribution to PCOS remains less well characterized than that of canonical WNT/β-catenin signaling. The WNT signaling pathway has also emerged as an important regulator of ovarian physiology and metabolic homeostasis. WNT signaling participates in ovarian folliculogenesis, granulosa-cell function, steroidogenesis and female reproductive development (79, 84, 85). Experimental studies demonstrate that WNT4/β-catenin signaling is required for normal follicular development and steroidogenic function (84). Alterations in WNT-related signaling have also been reported in PMOS, although the precise contribution of individual WNT ligands appears to be context- and tissue-dependent (8, 86). In particular, altered expression of WNT3, WNT7A and WNT8B has been investigated in reproductive tissues of women with PMOS (86). In T2DM, WNT ligands and their downstream signaling pathways have been implicated in glucose metabolism, adipose-tissue biology, pancreatic β-cell function and diabetes-related complications (87). Collectively, these findings indicate that dysregulated WNT signaling may represent another molecular intersection between reproductive and metabolic dysfunction, although further human studies are required to define its therapeutic relevance.

The strategies targeting these shared molecular mechanisms of PMOS and T2DM have shown considerable clinical benefit. Among these, metformin remains an important insulin-sensitizing agent because it improves insulin sensitivity and reduces hepatic gluconeogenesis in T2DM; in selected women with PMOS, it may improve metabolic parameters, menstrual cyclicity and androgen-related abnormalities, particularly in those with IR or metabolic risk (73, 74). Thiazolidinediones, through activation of peroxisome proliferator-activated receptor-γ (PPAR-γ), improve insulin sensitivity and have demonstrated effects on hyperandrogenism and ovulatory dysfunction in PMOS (88). Anti-androgen therapies such as spironolactone may be used to manage clinical manifestations of hyperandrogenism, particularly hirsutism, although they do not directly correct the underlying metabolic abnormalities. Lifestyle modification, including dietary improvement, regular physical activity and weight management, remains a cornerstone of management because it can improve metabolic health and several reproductive features of PMOS (89).

Emerging therapeutic approaches are increasingly focused on molecular targets implicated in the pathophysiology of both disorders. Modulation of WNT signaling has attracted interest because of its involvement in ovarian function, folliculogenesis, glucose metabolism and diabetes-related complications; however, WNT-directed therapy remains investigational and should not currently be presented as an established treatment for PMOS or T2DM (87). Similarly, anti-inflammatory strategies targeting pathways such as NF-κB and cytokine signaling have potential but require further clinical validation before routine therapeutic application (76, 77). Antioxidant-based approaches, including vitamin D, omega-3 fatty acids and N-acetylcysteine, have also been investigated for their potential effects on oxidative stress, inflammation and insulin sensitivity, although the strength and consistency of clinical evidence vary among interventions. Collectively, these findings support an integrated approach targeting metabolic, inflammatory, oxidative and endocrine abnormalities in women with PMOS who are at increased risk of T2DM.

### 3.14 Therapeutic implications

Therapeutic strategies for metabolic and reproductive management of PMOS and T2DM are as follows:

#### 3.14.1 Established clinical interventions

Lifestyle modification remains the foundational first-line approach for managing both the metabolic and reproductive features of polycystic ovary syndrome (PCOS) and its frequent overlap with type 2 diabetes mellitus (T2DM). Multicomponent interventions that combine dietary energy restriction, increased physical activity and behavioral strategies consistently improve weight, central adiposity, IR, lipid profiles and menstrual cyclicity. The 2023 International Evidence-based Guideline for the Assessment and Management of PCOS emphasizes that healthy lifestyle behaviors should be recommended for all women with PCOS to optimize general health, body composition and weight management, with no single dietary or exercise regimen shown to be superior (90).

Weight management is particularly critical because even modest reductions (5–10% of body weight) can restore ovulation, reduce hyperandrogenism and improve metabolic parameters. In high-risk populations, intensive lifestyle programs have demonstrated long-term reductions in T2DM incidence (approximately 58% relative risk reduction in the Diabetes Prevention Program), underscoring their dual benefit in PCOS and T2DM (91).

Insulin-sensitizing pharmacotherapy, principally metformin, is the most firmly established pharmacological adjunct. Metformin improves IR, lowers fasting glucose and BMI, improves lipid profiles and can restore menstrual cyclicity, particularly in women with BMI ≥ 25 kg/m². Systematic reviews and meta-analyses of randomized controlled trials confirm modest but clinically relevant reductions in BMI, HOMA-IR and androgens and an increased likelihood of clinical pregnancy compared with placebo. The 2023 guideline recommends metformin primarily for metabolic features and considers it an efficacious adjunct to lifestyle intervention (92).

Other established pharmacological strategies include combined oral contraceptive pills (first-line for menstrual irregularity and clinical hyperandrogenism) and, when fertility is desired, letrozole (preferred first-line ovulation induction agent). Anti-androgens and mechanical hair-reduction techniques may be added for hirsutism when other measures are insufficient (90).

#### 3.14.2 Emerging pharmacological approaches

Newer metabolic and endocrine agents originally developed for T2DM and obesity are being actively evaluated in PCOS. Glucagon-like peptide-1 (GLP-1) receptor agonists (liraglutide, semaglutide, exenatide) produce clinically meaningful weight loss, improve insulin sensitivity and may favorably affect free androgen index and menstrual frequency. Network meta-analyses and systematic reviews indicate superior anthropometric benefits compared with metformin alone, although gastrointestinal side-effects are common and long-term reproductive safety data remain limited. The 2023 guideline therefore allows consideration of GLP-1 receptor agonists (and orlistat) for weight management according to general-population recommendations, with concurrent contraception advised (90).

Sodium-glucose cotransporter-2 (SGLT2) inhibitors (empagliflozin, dapagliflozin, canagliflozin) reduce body weight, HOMA-IR and androgen levels in overweight/obese women with PCOS. Meta-analyses show improvements in total testosterone, free androgen index and glycemic indices, with a more favorable gastrointestinal tolerability profile than metformin or GLP-1 agonists in some comparisons. Combination regimens of GLP-1 receptor agonists plus SGLT2 inhibitors appear to yield additive weight-loss benefits (93).

Inositols (myo-inositol ± D-chiro-inositol) are widely used as over-the-counter insulin sensitizers. They offer modest metabolic and ovulatory benefits with a favorable safety profile, yet the 2023 guideline notes limited clinical superiority over metformin for hirsutism or central adiposity and therefore positions them as an optional adjunct based on individual preference (90).

#### 3.14.3 Investigational molecular targets

At the molecular level, several signaling networks that contribute to IR, ovarian steroidogenesis, inflammation and follicular arrest are under active investigation.

##### 3.14.3.1 PI3K/Akt pathway

Impaired insulin-mediated activation of the phosphatidylinositol 3-kinase/protein kinase B (PI3K/Akt) cascade in skeletal muscle, adipose tissue and ovarian granulosa cells is a core feature of PCOS-related IR. Experimental restoration of PI3K/Akt signaling improves glucose uptake (via GLUT4 translocation) and may reduce androgen excess; however, direct pharmacological activators remain preclinical (44).

##### 3.14.3.2 AMPK/mTOR axis

AMP-activated protein kinase (AMPK) activation (the primary mechanism of metformin and of natural compounds such as berberine) suppresses the mechanistic target of rapamycin (mTOR) complex, thereby improving energy homeostasis, reducing granulosa-cell autophagy and attenuating hyperandrogenism. Dual AMPK/mTOR modulators and berberine–metformin combinations show promising preclinical and early clinical metabolic and reproductive effects, but require further validation (94).

##### 3.14.3.3 WNT/β-catenin signaling

Dysregulated WNT/β-catenin activity contributes to abnormal folliculogenesis, steroidogenesis and inflammation in PCOS. Both classical and non-classical WNT ligands (e.g., WNT5A) influence granulosa-cell proliferation, apoptosis and androgen production via cross-talk with PI3K/Akt. Small-molecule and natural inhibitors of this pathway are being explored experimentally, yet clinical translation remains distant (44).

##### 3.14.3.4 Inflammatory and steroidogenic networks

Chronic low-grade inflammation (NF-κB, NLRP3 inflammasome) and over-expression of steroidogenic enzymes (CYP17A1, StAR) amplify IR and hyperandrogenism. Agents that simultaneously dampen these pathways (certain natural compounds, selective androgen-synthesis inhibitors) are under early investigation (44).

Collectively, while lifestyle intervention and metformin remain the evidence-based foundation for metabolic and reproductive management of PCOS/T2DM, GLP-1 receptor agonists and SGLT2 inhibitors represent the most advanced emerging options. Molecular targets within the PI3K/Akt, AMPK/mTOR and WNT/β-catenin pathways offer mechanistic promise but are still largely experimental and require rigorous clinical validation before routine use.

## 4 Discussion

The present study highlights the substantial mechanistic convergence between PMOS and T2DM, suggesting that these disorders are not independent reproductive and metabolic diseases. Rather, they represent clinically distinct manifestations of a broader network of metabolic, inflammatory, endocrine and intracellular signaling abnormalities. IR, compensatory hyperinsulinemia, chronic low-grade inflammation, OS, adipose tissue dysfunction and altered PI3K/Akt, MAPK, AMPK–mTOR and WNT/β-catenin signaling provide important connections between metabolic dysfunction and reproductive abnormalities in PMOS. This interpretation is consistent with epidemiological evidence demonstrating a substantially increased risk of dysglycemia and T2DM among women with PMOS. A meta-analysis of 23 observational studies involving more than 319,000 participants reported an approximately 3.5-fold higher risk of T2DM among women with PMOS, although the magnitude of risk was strongly influenced by overweight and obesity (2, 3).

IR represents one of the most important interfaces between PMOS and T2DM. Under physiological conditions, insulin receptor activation induces IRS phosphorylation and subsequent activation of the PI3K/Akt pathway, culminating in GLUT4 translocation and glucose uptake in skeletal muscle and adipose tissue. In IR states, defects at the receptor and post-receptor levels, particularly aberrant IRS phosphorylation and impaired PI3K/Akt activation compromise the metabolic actions of insulin. Therefore, IR provides a plausible explanation for the simultaneous occurrence of impaired glucose metabolism and increased ovarian steroidogenesis in PMOS (7, 10, 13–16). Importantly, IR in PMOS cannot be attributed exclusively to obesity. Although obesity substantially amplifies metabolic risk, metabolic abnormalities have also been documented in non-obese women with PMOS. A systematic review and meta-analysis reported significantly increased odds of IR, impaired glucose tolerance and T2DM even among non-obese women with PMOS compared with non-obese controls (3). These observations support the concept that intrinsic abnormalities in insulin signaling, in addition to adiposity-related mechanisms, contribute to the metabolic phenotype of PMOS. The relationship between PMOS and T2DM is therefore likely to be bidirectional.

An important distinction between PMOS and T2DM is the reproductive consequence of hyperinsulinemia. Elevated insulin concentrations can enhance ovarian androgen biosynthesis and potentiate the steroidogenic effects of LH, particularly within theca cells. In parallel, hyperinsulinemia suppresses hepatic production of sex hormone-binding globulin (SHBG), increasing circulating free androgen concentrations. The resulting hyperandrogenic state contributes to abnormal follicular development, anovulation and menstrual dysfunction (4, 17–20). Hyperinsulinemia can increase androgen production, while androgen excess may itself contribute to adipose dysfunction and adverse metabolic signaling. The clinical importance is evidenced that women with PMOS have an increased risk of impaired glucose tolerance and T2DM compared with women without PMOS. However, the magnitude of this association varies according to obesity, ethnicity and the method used to assess glucose metabolism (2, 3).

The AMPK–mTOR axis provides another important molecular framework linking energy sensing with metabolic and reproductive physiology. Reduced AMPK activity together with inappropriate mTOR activation promotes a metabolic environment characterized by impaired insulin sensitivity, altered lipid metabolism and mitochondrial dysfunction (21–26). The therapeutic relevance of this pathway is particularly evident in the action of metformin, which activates AMPK and suppress mTOR signaling, although AMPK-independent mechanisms may also contribute to these effects (25, 26).

Chronic low-grade inflammation represents another major component of the shared pathophysiology of PMOS and T2DM. Excess nutrient availability, adipocyte dysfunction, elevated free fatty acids and hyperglycemia can activate inflammatory pathways, particularly NF-κB, leading to increased production of TNF-α, IL-6 and other inflammatory mediators (27, 29–35). These cytokines can interfere with insulin signaling by promoting inhibitory serine phosphorylation of IRS proteins and thereby contribute to impaired PI3K/Akt activation. The relationship between inflammation and IR is not unidirectional. IR and associated metabolic stress can themselves increase inflammatory signaling, establishing a self-reinforcing cycle. TNF-α, for example, can impair insulin signaling while promoting lipolysis and increasing circulating free fatty acids. IL-6 can contribute to altered glucose metabolism and stimulate hepatic acute-phase responses, including production of CRP (33–35). Consequently, inflammation may function as both a mediator and amplifier of metabolic dysfunction. OS further intensifies this pathological network. Hyperglycemia, elevated free fatty acids, mitochondrial dysfunction and activation of ROS producing enzymes increase intracellular reactive oxygen species. Excessive ROS can activate stress-sensitive pathways including JNK, p38 MAPK and NF-κB, while JNK-mediated inhibitory phosphorylation of IRS-1 can further impair insulin signaling (36–38). The resulting interaction between ROS generation and inflammatory signaling establishes a positive-feedback loop that may contribute to persistent IR and cellular dysfunction. Recent literature also emphasizes the interconnected nature of IR, inflammation, OS and WNT/β-catenin dysregulation in PMOS (7). Thus, rather than treating OS and inflammation as secondary consequences, they may be considered important mechanistic components of the metabolic reproductive network.

The WNT/β-catenin pathway represents an important and comparatively less established component of the PMOS–T2DM signaling network. Canonical WNT signaling regulates cellular proliferation, differentiation, tissue homeostasis and ovarian follicular development. In the ovary, appropriate WNT activity is required for granulosa cell proliferation, follicular maturation and normal ovarian function. Dysregulation of WNT signaling may consequently contribute to follicular arrest and abnormal ovarian morphology (42–44). The significance of WNT signaling extends beyond ovarian biology. WNT pathways are involved in adipocyte differentiation, glucose metabolism and interactions with insulin signaling networks. Consequently, altered WNT activity may provide a molecular connection between ovarian dysfunction and metabolic abnormalities. Moreover, much of the evidence connecting individual WNT components with PMOS originates from ovarian tissue, cell culture or experimental models. Recent published literature emphasize the complexity of WNT signaling in PMOS and the need for further mechanistic validation before specific WNT components can be considered as therapeutic targets (42–45).

Although IR and metabolic inflammation constitute major components of PMOS pathophysiology, reproductive dysfunction cannot be fully explained by peripheral metabolic abnormalities. Altered hypothalamic–pituitary–ovarian (HPO) axis activity, including increased GnRH pulsatility and preferential LH secretion, contributes to hyperinsulinemia, excessive ovarian androgen production and abnormal follicular development (46–51). Hyperinsulinemia may in reverse increase ovarian sensitivity to gonadotropic stimulation and potentiate theca cell androgen synthesis, while hyperandrogenism may further aggravate metabolic dysfunction. Thus, reproductive endocrine abnormalities and metabolic IR should be considered components of a mutually reinforcing network rather than isolated abnormalities.

Adipose tissue is increasingly recognized as an endocrine organ that actively participates in the pathogenesis of both metabolic and reproductive dysfunction. Adiponectin, leptin and resistin represent important mediators of this adipose–ovarian crosstalk. Reduced adiponectin is associated with IR and has been consistently reported in PMOS, whereas elevated leptin and resistin may contribute to inflammatory signaling and metabolic dysfunction (53–61). Adiponectin is particularly relevant because it activates AMPK and promotes fatty-acid oxidation, thereby improving insulin sensitivity. Reduced adiponectin may therefore contribute to the metabolic phenotype of PMOS while simultaneously intersecting with the AMPK–mTOR axis. Meta-analytic evidence supports lower adiponectin concentrations in women with PMOS, reinforcing its potential role in the metabolic component of the syndrome (53, 54). Leptin and resistin may provide complementary mechanisms. Hyperleptinemia in obesity and PMOS may reflect leptin resistance and can interact with hypothalamic and inflammatory pathways, whereas resistin has been implicated in inflammatory signaling and IR. PI3K/Akt dysfunction impairs metabolic insulin action; relative preservation of MAPK signaling may maintain or enhance steroidogenic responses; reduced AMPK activity and increased mTOR signaling disturb cellular energy balance; NF-κB-mediated inflammation and ROS activate stress pathways that further inhibit insulin signaling; and WNT/β-catenin dysregulation potentially integrates ovarian, adipose and metabolic dysfunction.

This integrated model also helps explain why PMOS is associated with a substantial long-term metabolic burden. Meta-analyses have demonstrated increased risks of metabolic syndrome and T2DM among women with PMOS, although obesity and other metabolic characteristics account for an important proportion of the observed risk (2, 3). Pharmacological insulin sensitization, particularly with metformin, may provide benefits across both metabolic and reproductive domains (9, 25, 26). However, emerging targets such as inflammatory signaling, OS and WNT pathways require considerably more evidence before they can be incorporated into routine clinical practice. The therapeutic implications should also be interpreted within the limitations of the current evidence base. Many molecular mechanisms described in the literature are derived from animal models, cultured cells or cross sectional human studies. A recent scoping review of molecular mechanisms of IR in PMOS similarly concluded that evidence for several proposed pathways remains tissue specific and that human mechanistic studies are comparatively limited (9). Defining these interactions more precisely may facilitate biomarker discovery and the development of targeted, phenotype-specific interventions aimed at reducing both reproductive morbidity and long-term cardiometabolic risk.

## 5 Conclusion

PCOS and T2DM mellitus exhibit substantial pathophysiological overlap, characterized by interconnected disturbances in insulin signaling, glucose and lipid metabolism, chronic low-grade inflammation, OS and endocrine regulation. IR and compensatory hyperinsulinemia constitute important mechanistic links between metabolic dysfunction and reproductive abnormalities in PMOS, while shared alterations in intracellular signaling pathways may further contribute to the development and progression of metabolic disease. The frequent coexistence of PMOS and T2DM therefore reflects a complex bidirectional interaction between metabolic and reproductive dysfunction rather than two independent pathological processes. A comprehensive understanding of these convergent molecular mechanisms may facilitate the identification of novel therapeutic targets and support the development of integrated, mechanism-based interventions addressing both metabolic and reproductive abnormalities. Such approaches may ultimately improve long-term cardiometabolic and reproductive outcomes in women with PMOS who are at increased risk of T2DM.
